# Effect of Natural Food Product Interventions on Chronic Diseases

**DOI:** 10.3390/foods13060849

**Published:** 2024-03-11

**Authors:** Tao Wu

**Affiliations:** State Key Laboratory of Food Nutrition and Safety, Food Biotechnology Engineering Research Center of Ministry of Education, College of Food Science and Engineering, Tianjin University of Science & Technology, Tianjin 300457, China; wutao@tust.edu.cn

With the rise in people’s living standards, chronic diseases such as obesity, diabetes and cardiovascular diseases have become predominant. These threaten human health, and thus people are paying more attention to the prevention of such diseases through food intervention. Therefore, the application of natural foods in the prevention of chronic diseases, such as polyphenols, polysaccharides, peptides and probiotics, has become a hot topic of attention.

In order to regulate human blood lipids and reduce the incidence of complications related to lipid metabolism disorders such as obesity, adjusting the consumption of different foods is a novel and feasible strategy. For example, through the collection and synthesis of clinical evidence, after meta-analysis, it was found that the intake of edible oil-derived MUFA (monounsaturated fatty acids) can increase the level of HDL-C (high-density lipoprotein cholesterol) in the blood. However, there was no effect on total cholesterol (TC), triglyceride (TG) or low density lipoprotein cholesterol (LDL-C) levels. The consumption of other food-derived mono-unsaturated fatty acids (ODMs) significantly decreased TG concentrations but did not affect the level of TC, LDL-C, or HDL-C. It is suggested that foods rich in MUFA may be beneficial to regulate the blood lipid profile [1].

Three research projects further explore natural foods by focusing on specific functional components such as Red-Skin Extracts of Lotus Seeds (RSEs), Coix Seed Oil (CSO), and Modified Highland Barley (MHB). These research works delve into their potential mechanisms and application prospects in more detail, supported by relevant instrumental measurements and data. For example, the composition of red skin Extracts of Lotus Seeds (RSEs) was preliminary identified using the UPLC-Q/TOF-MS system. In addition, in mouse feeding experiments, they found that RSE can reduce fat accumulation, up-regulate the activity and expression of LPL in skeletal muscle, and down-regulate the activity and expression of LPL in the adipose tissue of the epididymis by regulating the activity of tissue-specific lipoprotein lipase (LPL). This ultimately leads to weight loss [2]. The study mainly used supercritical fluid extraction of CSO and found that CSO can improve the liver lipid deposition and lipid metabolism caused by a high-fat diet [3]. In addition, medium chain fatty acids rich in CSO can effectively regulate liver fatty acid metabolism and the lipid levels of obese mice induced by a high-fat diet. Another study made a great contribution to the aspects of lipid metabolism and liver damage regulation in mice with a high-fat and high-cholesterol diet (HFCD).

Three kinds of improved highland barley (MHB), namely microwave fluidized HB, extruded and puffed HB, and ultrafine pulverized HB, increased the Bacteroidetes/Firmicutes ratio and the abundance of Lactobacillus and heterobacterium while decreasing the abundance of Proteobacteria, which is related to lipid-metabolizing bacteria [4]. Liquid chromatograph mass spectrometer metabolomics studies showed that MHB supplementation significantly increased the level of metabolites and promoted the activation of the arachidonic acid metabolic pathway, the expression of ABC transporter, bile secretion, primary bile acid biosynthesis, and so on.

In addition, a growing body of research shows that the gut microbiota is one of the key factors that may influence nutrient metabolism and immune response. Therefore, in a study of lipid metabolism-related disorders, besides measuring lipid-related characteristics, increasing beneficial effects of the gut microbiota have been described. For example, on the basis of a kind of buckwheat–oat–pea composite flour (BOP; quality ratio of buckwheat–oats–peas = 6:1:1), the rat diabetes model was established by a high-fat diet and streptozotocin injection. It was found that BOP could significantly change the glucose and lipid metabolism of diabetic rats, alleviate liver injury, and change the composition of the gut microbiota, making it a stable food substitute for preventing diabetes [5].

Large yellow tea water extract (LWE) supplement can suppress lipogenesis and regulate gut microbiota through the SIRT 6/SREBP 1 pathway, increase gut microbiota diversity, produce short-chain fatty acid (SCFA) microbiota, and increase SCFA concentration. Overall, it can prevent and improve the metabolic syndrome of male leptin receptor knockout (Lepr^−/−^) rats [6].

Gastric ulcer (GU) is one of the most common digestive diseases in the world. Natural plant polysaccharides can inhibit IBD by regulating intestinal flora and metabolism [7]. This study is the first to explore the therapeutic effect of *Sarcodon aspratus* polysaccharides (SAFP) on GU induced by water immersion and restraint stress (WIRS) in rats. At the same time, the beneficial effects of SAFP on intestinal flora homeostasis in GU rats were also studied, providing data support for SAFP as a natural gastric mucosal protective agent and a potential health product to prevent GU and its related complications. In this study, we also found that gut microbiota plays an important role. SAFP regulates the ecological imbalance of gut microbiota by increasing the relative abundance of probiotics and reducing the bacterial proliferation triggered by WIRS.

After cooking yam samples in seven different ways, without adding additives and seasonings, the regulatory effects of yam gut microbiota and its non-starch polysaccharides (NSPs) were evaluated through in vitro digestion and fermentation protocols. Studies have found that yam polysaccharides can enhance the relative abundance of probiotics (such as macrococcus and bifidobacterium) while reducing the number of pathogenic bacteria [8]. In addition, yam polysaccharides can also increase the level of production of short-chain fatty acids.

The regular consumption of high-energy-density foods that are high in saturated fat and low in fiber can lead to the development of metabolic abnormalities. To address this issue, a review in this study explores the impact of inulin, a soluble dietary fiber commonly found in chicory root, as a replacement for sucrose in rats fed a high-fat (HF) diet [9]. Inulin is not easily digested in the gut and has been shown to have beneficial effects on fecal microbiota, cardiometabolic risk factors, eicosanoids, and oxidative stress. This study lays the groundwork for further research into the potential mechanisms by which inulin may influence cardiometabolic outcomes.

Additionally, it was found in another review that the immunomodulatory properties of polyphenols may help to reduce cardiovascular risk [10]. The researchers investigated the protective effects of polyphenol extract (PE), specifically its two phenolics brachythol B (BB) and ethyl gallate (EG), on foam cell formation and the inflammation of RAW264.7 macrophages induced by oxidized low-density lipoprotein (ox-LDL).

Two review articles were also included in this Special Issue, one of which examined the inhibitory effect of silkworm chrysalis protein hydrolysate (SPPHs) on the proliferation of various malignant tumor cell types, including gastric cancer cells [11]. It was further demonstrated that this inhibitory effect was dose-dependent. Another paper outlined the development of a novel walnut peptide chelate (WP-Fe). In this study, they used molecular docking technology to establish a binding model of ferrous ion and WP based on low-molecular-weight walnut peptide (WP). The chelation rate of WP-Fe formed by this method could reach 71.87 ± 1.60%. The iron content is as high as 113.11 ± 2.52 mg/g, so this WP-Fe is expected to become a dual nutritional supplement alongside iron and antioxidants, and it also lays the foundation for further cell and in vivo absorption experiments [12].

Together, these articles expand applied knowledge on the intervention role of natural foods in chronic diseases and promote the development of functional ingredients in the food industry.
